# Factors Related to Sustained Use of a Free Mobile App for Dietary Self-Monitoring With Photography and Peer Feedback: Retrospective Cohort Study

**DOI:** 10.2196/jmir.3084

**Published:** 2014-04-15

**Authors:** Elina Helander, Kirsikka Kaipainen, Ilkka Korhonen, Brian Wansink

**Affiliations:** ^1^Department of Signal ProcessingTampere University of TechnologyTampereFinland; ^2^VTT Technical Research Centre of FinlandTampereFinland; ^3^Charles H. Dyson School of Applied Economics and ManagementCornell UniversityIthaca, NYUnited States

**Keywords:** healthy eating, food journaling, food photographing, adherence, mobile app, self-monitoring, peer feedback, control theory

## Abstract

**Background:**

Healthy eating interventions that use behavior change techniques such as self-monitoring and feedback have been associated with stronger effects. Mobile apps can make dietary self-monitoring easy with photography and potentially reach huge populations.

**Objective:**

The aim of the study was to assess the factors related to sustained use of a free mobile app (“The Eatery”) that promotes healthy eating through photographic dietary self-monitoring and peer feedback.

**Methods:**

A retrospective analysis was conducted on the sample of 189,770 people who had downloaded the app and used it at least once between October 2011 and April 2012. Adherence was defined based on frequency and duration of self-monitoring. People who had taken more than one picture were classified as “Users” and people with one or no pictures as “Dropouts”. Users who had taken at least 10 pictures and used the app for at least one week were classified as “Actives”, Users with 2-9 pictures as “Semi-actives”, and Dropouts with one picture as “Non-actives”. The associations between adherence, registration time, dietary preferences, and peer feedback were examined. Changes in healthiness ratings over time were analyzed among Actives.

**Results:**

Overall adherence was low—only 2.58% (4895/189,770) used the app actively. The day of week and time of day the app was initially used was associated with adherence, where 20.28% (5237/25,820) of Users had started using the app during the daytime on weekdays, in comparison to 15.34% (24,718/161,113) of Dropouts. Users with strict diets were more likely to be Active (14.31%, 900/6291) than those who had not defined any diet (3.99%, 742/18,590), said they ate everything (9.47%, 3040/32,090), or reported some other diet (11.85%, 213/1798) (χ^2^_3_=826.6, *P*<.001). The average healthiness rating from peers for the first picture was higher for Active users (0.55) than for Semi-actives (0.52) or Non-actives (0.49) (*F*_2,58167_=225.9, *P*<.001). Actives wrote more often a textual description for the first picture than Semi-actives or Non-actives (χ^2^_2_=3515.1, *P*<.001). Feedback beyond ratings was relatively infrequent: 3.83% (15,247/398,228) of pictures received comments and 15.39% (61,299/398,228) received “likes” from other users. Actives were more likely to have at least one comment or one “like” for their pictures than Semi-actives or Non-actives (χ^2^_2_=343.6, *P*<.001, and χ^2^_2_=909.6, *P*<.001, respectively). Only 9.89% (481/4863) of Active users had a positive trend in their average healthiness ratings.

**Conclusions:**

Most people who tried out this free mobile app for dietary self-monitoring did not continue using it actively and those who did may already have been healthy eaters. Hence, the societal impact of such apps may remain small if they fail to reach those who would be most in need of dietary changes. Incorporating additional self-regulation techniques such as goal-setting and intention formation into the app could potentially increase user engagement and promote sustained use.

## Introduction

### Dietary Self-Monitoring and Feedback

Despite various efforts to curb the growth of obesity, a significant part of the population still eats unhealthy food in excessive quantities. Knowledge about healthy eating is not sufficient on its own to change eating behavior [1]. On an individual level, one behavioral strategy recommended in weight control and improvement of dietary habits is self-monitoring of food intake [2,3]. Moreover, healthy eating interventions that use self-monitoring combined with other self-regulation techniques from control theory, such as feedback [4], appear to be more effective than interventions that do not include these techniques [5]. Smartphones and photography can be used to make self-monitoring easy and convenient [6-9]. Due to the wide penetration of smartphones in the population, this approach could reach a large number of people with small cost. However, it is not known whether such mobile apps for independent use would engage people and reach those who could benefit the most from dietary monitoring.

Dietary self-monitoring prompts people to reflect on their current behavior and compare it to ideal behavior [4,10]. In weight loss studies, consistent recording of food intake appears to be one of the most effective methods [3,11]. Yet it is not clear how consistent self-monitoring needs to be for the method to be effective. The degree of monitoring is typically reported as the number of food diaries/entries completed per day and/or week, and the duration of the monitoring period has varied from eight weeks to two years in different studies [3]. For example, a six-month intervention study found that the average number of food records per week was 3.7 and greater weight loss was associated with more frequent monitoring [12]. Another study on members of a free online weight loss program found that more frequent weight monitoring was associated with greater weight loss, but no association was found between dietary monitoring frequency and weight loss [13]. Overall, it is not clear whether these results tell more about an individual’s engagement to the program or specifically about the effect of monitoring. Studies have also mostly focused on weight loss, not on prevention of weight gain through improvement of eating behavior.

Traditional methods for dietary self-monitoring include more or less detailed food diaries and calorie counting [14]. These methods can be burdensome to people [6,7,15-17] and suffer from underreporting and recall issues [15-19]. Methods that can minimize the temporal distance between eating and recording food intake are likely to improve outcomes; the percentage of food records made within 15 minutes of eating has been found to be associated with weight loss [20]. Recently, smartphone cameras have made just-in-time food journaling possible by taking a photo of food. A pilot study using disposable cameras suggests that recording food before eating can lead to increased consideration of dietary habits and alter food choices better than written diaries [21]. Capturing images of food may improve adherence and accuracy in some groups, such as among adolescents who may be less motivated to keep detailed food diaries [8].

Feedback on performance is a self-regulation technique that either reinforces the current behavior or creates a discrepancy between current and ideal behavior [4]. Individualized feedback has been found to be associated with higher adherence to online interventions promoting healthy lifestyles [22,23]. In terms of the content of feedback, encouraging reflection and self-monitoring may be more important than detailed analysis of nutritional contents when the target is to change eating behavior [10,24,25].

Mobile apps can provide automated feedback on the healthiness of the food based on the photo and also leverage other users to provide feedback through crowdsourcing [6]. Although it is still difficult to estimate food ingredients and portion sizes from a photograph, efforts to develop estimation algorithms based on image processing or crowdsourcing are underway [6,16]. One such app is PlateMate, which crowdsources nutritional assessments from Amazon Mechanical Turk, where individuals assessing the food pictures receive a nominal payment for each picture [6]. Evaluation of the app suggested that these crowd-generated assessments were almost as accurate as those done by professional nutritionists, although pictures containing ambiguous items such as beverages or salad dressing received inaccurate ratings [26]. Beyond nutritional assessments, technology can be used to share advice and feedback on healthy eating between users [27].

### Adherence to Mobile Apps

Numerous mobile apps for healthy eating are available in application markets. Although they are easily within anyone’s reach, attrition is likely to be a significant challenge, since there is usually very little external pressure or incentive to continue usage [28]. Little research exists about usage behavior of health-promoting apps, but reviews on Web-based interventions have found that adherence is generally lower outside randomized controlled trials and some observational studies have reported adherence rates as low as 1% [29]. One of the few articles published about mobile app usage examined data that was collected from 4125 users between August 2010 and January 2011 [30]. The study found that sessions with apps were short, averaging a little more than a minute, and that different types of apps were used during different times of the day. Communication apps were the most frequently used, 49.50% of app launches, whereas the proportion of health apps was only 0.26% of all app launches [30]. More studies into health app usage behavior are thus warranted. Considering that people’s eating patterns and daily routines vary over the week [31,32], analyzing the temporal context of app usage may help identify the best times to start using an app that promotes lifestyle changes.

### Study Objectives

This study assesses the overall usage and reach of a free mobile app for healthy eating (“The Eatery”) over a period from October 2011 to April 2012. Specifically, we examine the indicators of sustained use of the app, especially focusing on the initiation of self-monitoring and the influence of peer feedback.

## Methods

### Mobile App

**Figure 1:**
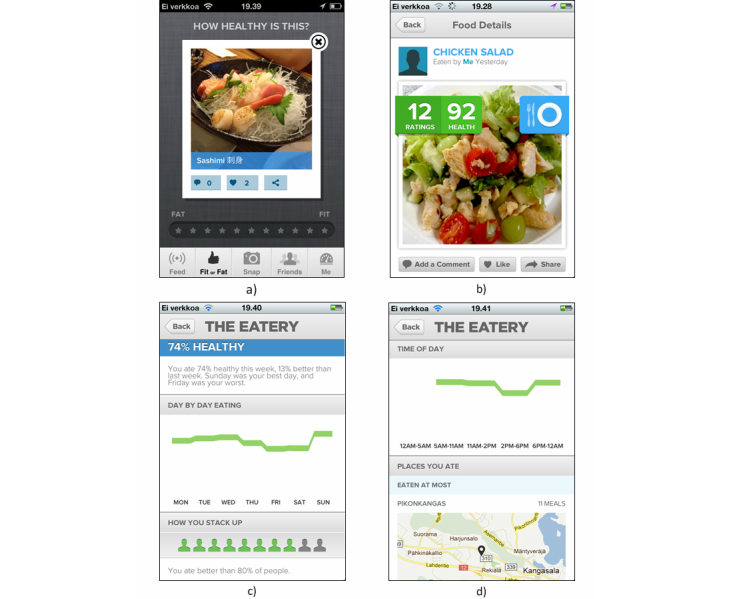
Screenshots of The Eatery app: a) rating other people’s food with fat-fit scale, b) feedback received for photographed food, c) weekly summary, and d) summary of user’s time-of-day healthiness ratings and places eaten at most.

The Eatery was a free iPhone app developed by the company Massive Health. The app was officially launched on November 1, 2011 in Apple’s application market [33]. It was targeted toward English-speaking people and presented as an easy and fun way to eat healthily. Its main functions were photographic food recording, self-evaluation of foods, and crowdsourced peer feedback. Users were asked to take a picture of the foods they were going to eat, rate the picture on an arbitrary healthiness scale from fat (unhealthy) to fit (healthy) (Figure 1), and optionally write a description for the picture. In addition to self-evaluation, users were prompted to rate another user’s food pictures every time they opened the app. They could rate as many successive pictures as they wanted (Figure 1a). Each of the user’s own food pictures received an average healthiness rating that was calculated from ratings given by other users (Figure 1b) and displayed as a number between 0 (“fat”) and 100 (“fit”). Other social support features of the app included the option to follow other users and provide feedback for their pictures in the form of comments and “likes”. The app provided automated feedback on the user’s past eating behavior by showing the past week’s day-to-day healthiness ratings and overall rating (Figure 1c), allowing the user to note the worst times as potential improvement points. Feedback was also given on week-to-week progress in healthiness ratings and on social comparison to other users (Figure 1c). Moreover, the app provided information on past behavior by displaying the most frequent eating locations (Figure 1d) and highlighting the best and worst meals of the week. The only background information asked from the users was their dietary preference on the first launch of the app.

### Study Sample

Altogether 189,770 users downloaded and used the app, The Eatery, at least once between October 15, 2011, and April 3, 2012. During this time, they generated 429,288 pictures and 7,946,447 ratings. In May 2012, Massive Health, the developer of the app, decided to make the anonymized dataset available for research purposes upon contact. The authors obtained the dataset from Massive Health in June 2012.

Data of users, pictures, and ratings included timestamps that represented the local time of the user’s mobile phone. The timestamps that were stored when the user first used the app included time zone information for 98.51% (186,933/189,770) of the users: 68.41% (127,884/186,933) of them were from the main US time zones (UTC-8 to UTC-5) and 12.48% (23,335/186,933) were from the main European or African time zones (UTC+0 to UTC+3).

### Definition of Variables

Table 1 lists the variables used in the analyses of factors related to usage of the app. As the focus of this study is on dietary self-monitoring, the usage period was defined as the time that elapsed between the first and the last picture taken by the user, even though the users who stopped taking their own food pictures could still continue to rate other users’ pictures.

**Table 1:** Variables related to the usage of the mobile app “The Eatery”.

Category	Variable	Description
**Usage activity**		
	Number of pictures	Total number of pictures taken by the user
	Usage period	Time elapsed between the first picture and the last picture (ie, the duration of self-monitoring)
	Pictures per day	Average number of pictures the user took per day during the usage period
	Ratings given for peers	Total number of ratings the user gave for other users’ pictures
**Context of use**		
	Registration time	Day of week (Sun-Sat) and time of day when the user first used the app
	Dietary preference	The response the user gave to “How do you eat?” question during the first launch of the app. The preference categories are listed in Table 2.
**Self-evaluation**		
	Own healthiness rating	Healthiness rating the user gave for an own picture (0 to 1)^a^
	Picture description length	Number of characters written in the picture description
**Peer feedback**		
	Average healthiness rating	Mean peer rating given for the picture (0 to 1)^a^
	Number of ratings	Total number of peer ratings given for a picture
	Number of comments	Total number of comments from peers for a picture
	Number of likes	Total number of peers who “liked” a picture
	Difference to peer ratings	Difference between the user’s own healthiness rating and average healthiness rating for a picture

^a^Healthiness ratings were stored as a decimal number from 0 (“fat”) to 1 (“fit”), whereas the user saw the ratings as numbers from 0 to 100, as in Figure 1b. The rating that was displayed to the user was a non-linear mapping from peer ratings and user’s own rating.

### Exclusion Criteria in Analyses

Pictures that did not contain an actual image (3.13%, 13,433/429,288 were such “empty pictures”) were removed from the data, resulting in a sample of 415,855 pictures for analysis. The overall quality and content of the pictures was screened by the researchers by examining a random sample of pictures. The examination revealed that, for some users, the first picture served as a test picture (for example, they took a picture of a chair to test out the application). Further examination showed that pictures obtaining a low number of peer ratings were typically something other than food, and therefore should be removed from further analysis. By manual inspection, the threshold of a valid picture was adjusted to 10 ratings: if the first picture taken by a user had received less than 10 ratings, the picture information was excluded and the second picture was used instead. If the user had taken only one picture or the second picture had received less than 10 ratings, the user was excluded from the analyses concerning the peer feedback for the first picture. The total number of pictures for each user was adjusted after the picture validity check of the first two pictures. This decreased the total number of pictures by a user by two pictures at most. Latter pictures were not examined. In total, 398,228 pictures (92.76% of all pictures and 95.76% of non-empty pictures) were classified as valid pictures.

Some users had not rated their own first picture—these users were excluded when analyzing the difference between their own and average peer healthiness ratings.

### Adherence Levels

Individual users in the dataset were divided into groups based on their adherence, for the analysis of different indicators of adherence (initiation context of self-monitoring and peer feedback). The level of adherence was defined based on the total number of pictures taken and the length of the usage period of the app.

Two types of adherence classifications were formed for different analyses. In the first case, two user groups were formed: (1) users who had taken no valid pictures or only one valid picture (“Dropouts”, 86.39%, 163,949/189,770), and (2) users who had taken more than one valid picture (“Users”, 13.61%, 25,821/189,770). For users who had taken at least one valid picture, three activity levels were defined: (1) “Actives” who had taken at least 10 pictures and had used the app at least one week (2.58%, 4895/189,770), (2) “Semi-actives” who had taken at least two pictures, but less than 10 pictures or whose usage period was less than one week (11.03%, 20,926/189,770), and (3) “Non-actives” who had taken only one valid picture (17.36%, 32,948/189,770).

The proportion of users who had downloaded the app less than one week before the sampling period ended (on March 28, 2012 or later) was 2.01% (3812/189,770). Hence, they could not be classified as Actives. They were still included in the analyses due to their small number.

### Initiation of Self-Monitoring

The association between users’ registration time and adherence level was analyzed to determine whether the temporal context of initial use could have an influence on subsequent usage activity. Registration time was categorized into seven weekdays and each day was divided into five time intervals: time between 0-5 (night), 5-10 (morning), 10-15 (daytime), 15-19 (late afternoon), and 19-24 (evening). These time intervals were chosen to correspond to the natural periods of the day and based on the assumption that most users were from Anglo-American culture, since the app was in English and roughly 68% (127,884/186,933) of the users registered from the main US time zones. The number of Dropouts and Users who had started using the app on each weekday and time of day intervals were calculated. The chi-square (χ^2^) test was used to compare whether the proportions of Dropouts and Users in weekday and time of day intervals (35 options) were equal to each other. Bonferroni correction was used to adjust for multiple comparisons and the adjusted significance level was set at *P*=.0014. Further comparisons were exploratory and were made based on initial results. Registration time was not available for 1.49% of the users (2837/189,770).

Dietary preferences were divided into four categories based on the users’ response to “How do you eat?” question on the first use of the app. Table 2 lists the answer options to the question and the numbers of users in each category: (1) “Not defined” included users who had not given any preference (42.22%, 80,118/189,770), (2) “Everything” included users who chose the option “I eat everything” (46.33%, 87,912/189,770), (3) “Strict” included users who had at least one of the following options chosen: “Low carb, no carb, or paleo”, “Low fat”, or “Vegan/vegetarian”, (8.97%, 17,025/189,770), and (4) “Other” included users who had chosen or written an option that was not included in the first three classes (2.48%, 4715/189,770). For example, the variations of “I eat everything!” response such as “I eat everything except…” were categorized as “Other”.

The associations between dietary preferences and adherence were analyzed by calculating the proportion of (1) Actives out of Users + Non-actives (ie, out of all users who took at least one valid picture), and (2) Users out of Users + Non-actives for each dietary preference category. The chi-square test was used to examine whether the proportions were equal between different dietary preference categories. Tukey’s HSD (honestly significant difference) multiple comparison test among proportions was used to analyze which dietary preference categories differed from each other after obtaining significance value *P*<.05.

Finally, the existence and length of the textual description given for the first picture taken by the user were compared between Active, Semi-active, and Non-active user groups. One-way ANOVA (analysis of variance) was used for description length and the chi-square test for the existence of the description. This analysis was done to assess the engagement level of the user during the initiation of self-monitoring.

**Table 2:** Numbers of users according to their dietary preferences based on “How do you eat?” question.^a^

“How do you eat?”	Category	Number of users, n (%) n=189,770
Not defined	Not defined	80,118 (42.22%)
“I eat everything!”	Everything	87,912 (46.33%)
“Low fat”	Strict	7778 (4.10%)
“Low carbs, no carbs, or paleo”	Strict	7146 (3.77%)
“Vegan or vegetarian”	Strict	6223 (3.28%)
“Complex carb diet”	Other	2388 (1.26%)
“Other”	Other	2427 (1.28%)
“Gluten free” or “gluten free”	Other	229 (0.12%)
None of the above	Other	1714 (0.90%)
Total	Strict	17,025 (8.97%)
Total	Other	4715 (2.48%)

^a^Note that some users provided multiple responses to the question.

### Peer Feedback

The amount and quality of peer feedback given for the first picture (average healthiness score, number of likes, number of comments, and difference to peer ratings) were compared between Active, Semi-active, and Non-active user groups to determine whether higher level of feedback on the initiation of self-monitoring was connected with adherence. Only those who had at least one valid picture among the first two pictures they had taken were included because the focus was on the initial feedback. For continuous variables, one-way ANOVA was used and for binary variables, the chi-square test was used to compare whether the proportions were equal between user groups. The numbers of ratings given by the users in each dietary preference category were also calculated to determine whether the stated dietary preference would have a connection to the user’s activity in providing peer feedback to others.

### Changes in Healthiness Ratings

Changes in healthiness ratings were analyzed only among Active users who had at least one valid picture among the first two pictures they had taken (99.35% of Actives, 4863/4895). Other user groups used the app for such a short time that no trend could reliably be identified. First, a correlation coefficient between the average healthiness rating of the first picture and all subsequent pictures was determined. A change (linear regression coefficient) in healthiness ratings as a function of picture index and corresponding *P* value was calculated for each Active user. The dependent variable was the healthiness rating of a picture and the independent variable was the picture index 1,2,…,*N* where *N* was the number of pictures taken by the user. Note that the ordered list of pictures was used instead of real time axis. If a significant (*P*<.05) positive linear coefficient was found, the user was categorized into “Improvers” (improvement in diet), and negative into “Decliners” (deterioration in diet). Student’s *t* test was used to compare whether usage activity (number of pictures, usage period, and pictures per day) differed between Improvers and other Actives.

Changes in eating behavior among users with different dietary preferences were also examined. One-way ANOVA was used to compare the average healthiness rating for the first picture and the healthiness rating for all pictures between different dietary preference categories. The number of Improvers or Decliners in each dietary category was determined. The chi-square test was used to examine whether there were an equal proportion of Improvers and Decliners in each dietary preference category.

## Results

### Overall Adherence and Healthiness Ratings

Table 3 shows the numbers of users divided into different adherence levels based on their usage activity. The average number of pictures and usage period in days is also shown for Semi-actives and Actives. Only 2.58% (4895/189,770) of the users became Active users, whereas more than two-thirds of the users did not take any valid pictures. On average, Actives took 1.6 pictures per day and 14.99% (734/4895) of them took more than three pictures per day.

Table 4 summarizes the statistics of valid pictures (92.76% of all pictures, 398,228/429,288) in the dataset from The Eatery. Their average healthiness rating, 0.58, was slightly above the midpoint of the fat-fit scale from 0 to 1.

**Table 3:** Adherence data for users who downloaded the free dietary self-monitoring app between October 15, 2011 and April 3, 2012 (n=189,770).

User group	Activity level	Description	Count, n (%)	Pictures per user, mean (SD)	Usage period in days, mean (SD)
Dropouts	Non-users	No pictures or no valid pictures	131,001 (69.03%)	-	-
Dropouts	Non-actives	Only 1 valid picture	32,948 (17.36%)	-	-
Users	Semi-actives	At least two valid pictures and less than 10 pictures or usage period shorter than 7 days	20,926 (11.03%)	4.1 (3.7)	9.3 (19.2)
Users	Actives	At least 10 pictures and usage period longer than 7 days	4895 (2.58%)	58.9 (99.5)	46.6 (37.7)

**Table 4:** Statistics for the 398,228 valid pictures taken by 58,769 users of the dietary self-monitoring app “The Eatery”.

Variable	Description	Value, mean (SD; range) or n (%)
**Self-evaluations**		
	Number of pictures with textual description	293,692 (73.75%)
	Average length of textual description (if existed) as number of characters	26.1 (18.1; 1-248)
**Peer feedback**		
	Average healthiness rating	0.581 (0.195; 0.0261-0.986)
	Number of pictures having at least one like	61,299 (15.39%)
	Average number of likes (if existed)	1.3 (0.9; 1-21)
	Number of pictures having at least one comment	15,247 (3.83%)
	Average number of comments (if existed)	1.7 (1.4; 1-28)

### Initiation of Self-Monitoring

**Figure 2:**
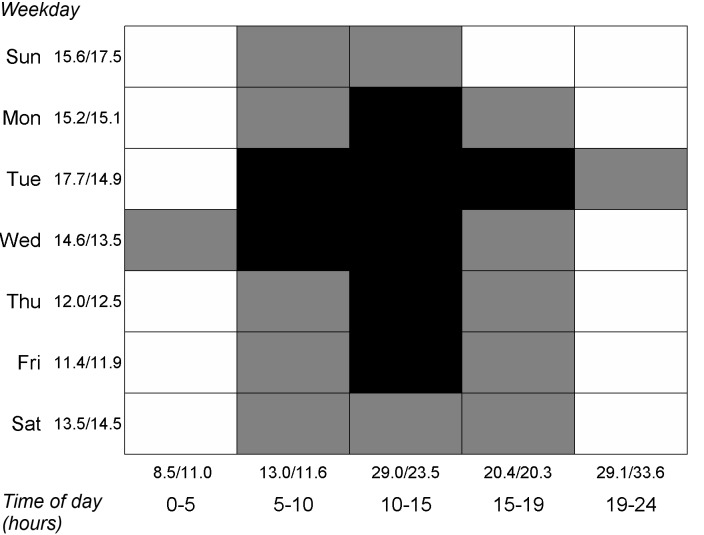
Correlations between users’ adherence level and their local registration time. Black=higher proportion of Users (*P*<.0014); White=higher proportion of Dropouts; Grey=no difference. Numbers separated by slashes next to weekday and time of day labels are percentages of Users/Drop-outs for corresponding rows and columns.

The associations between users’ registration time and adherence level are presented in Figure 2, which compares the proportions of Users and Dropouts who started using the app on each day of week and time of day interval. A higher proportion of Users started using the app during the daytime on weekdays than Dropouts (20.28%, 5237/25,820 vs 15.34%, 24,718/161,113; *χ* ^*2*^_*1*_=356.3, *P*<.001). Moreover, a higher proportion of Users started using the app especially during Tuesdays than Dropouts (17.66%, 4561/25,820 vs 14.88%, 23,974/161,113; *χ* ^*2*^_*1*_=133.4, *P*<.001).

Most common dietary preferences (see Table 2) reported by users during the first use of the app were “I eat everything” (46.33%) or undefined (42.22%). In total, 8.97% of the users were considered to have Strict diets. Table 5 presents the differences in adherence levels between dietary preference groups among users who took at least one valid picture. Users with Strict diets were the most likely (14.31%, 900/6291) and users who had not defined any diet were the least likely (3.99%, 742/18,590) to use the app actively. A similar trend was observed when Semi-active users were included in the comparisons: half (50.45%, 3174/6291) of the users with Strict diets were Semi-active or Active, a significantly higher proportion than among users who had not defined their diets.

Engagement of the user during the initiation of self-monitoring was also assessed by examining the textual description given for the first picture. A textual description for the first picture was given by 26.09% (15,179/58,170) of users who had at least one valid picture among the first two pictures they had taken. Table 6 presents the comparisons between different adherence groups. Active users had written a description for the first picture more often than Semi-actives or Non-actives, and the average description was also longer in the number of characters.

**Table 5:** Proportions of Semi-active and Active users in each dietary preference category out of all users who took at least one valid picture.

Users	1. Not defined, n (%) n=18,590	2. Everything, n (%) n=32,090	3. Strict, n (%) n=6291	4. Other, n (%) n=1798	Test statistics	Differences in post hoc comparisons
Actives / Users+Non-actives	742 (4.0%)	3040 (9.47%)	900 (14.31%)	213 (11.9%)	*χ* ^*2*^_3_=826.6, **P*<.*001	All groups
Users / Users+Non-actives	7188 (38.67%)	14,560 (45.37%)	3174 (50.45%)	899 (50.0%)	*χ* ^*2*^_3_=371.8, **P*<.*001	All but not 3 and 4

**Table 6:** Comparison of user engagement in the first self-monitoring entry between different adherence groups as measured by the presence and length of textual description for the picture.

First picture characteristics	1. Non-actives (n*=*32,648)	2. Semi-actives (n*=*20,659)	3. Actives (n*=*4863)	Test statistics	Differences in post hoc comparisons
Presence of textual description, n (%)	5783 (17.71%)	6824 (33.03%)	2572 (52.89%)	*χ* ^*2*^_2_=3515.1, **P*<.*001	All groups
Number of characters in description (if existed), mean (SD)	20.1 (15.8)	23.4 (17.5)	26.8 (19.1)	*F*_2,15176_=150.1, **P*<.*001	All groups

### Peer Feedback

Feedback received by the users’ first pictures was examined to determine whether higher level of feedback on the initiation of self-monitoring was connected with adherence. The first picture had at least one like among 7.68% (4470/58,170) of the users and at least one comment among 3.85% (2240/58,170) of them. Table 7 presents the comparisons of variables related to peer feedback between different adherence groups. Small but significant differences were found between Active and less active users for all variables: the average healthiness rating was higher and the proportion of pictures having comments and likes was higher. Still, comments and likes were relatively rare even among Active users.

Peer feedback was also examined from the perspective of users who gave the ratings to others. Analysis of dietary preferences and rating activity found that users in the “Not defined” diet group gave 21.81% (1,732,976/7,946,447) of all ratings, users in the “Everything” group gave 52.83% (4,198,272/7,946,447), users with Strict diets gave 20.70% (1,645,134/7,946,447), and users with some Other diet gave 4.66% (370,065/7,946,447) of all ratings.

**Table 7:** Amount and quality of peer feedback for the initial self-monitoring record in the app between different adherence groups.

First picture characteristics	1. Non-actives (n*=*32,648)	2. Semi-actives (n*=*20,659)	3. Actives (n*=*4863)	Test statistics	Differences in post hoc comparisons
Average healthiness rating, mean (SD)	0.49 (0.21)	0.52 (0.20)	0.55 (0.19)	*F*_2,58167_=225.9, **P*<.*001	All groups
Difference to peer ratings, mean (SD)	0.04 (0.22)	0.04 (0.21)	0.05 (0.18)	*F*_2,57738_=5.1, **P*=.*006	1 and 3, 2 and 3
Having at least one like, n (%)	2031 (6.22%)	1792 (8.67%)	647 (13.30%)	χ^2^_2_=343.6, **P*<.*001	All groups
Number of likes (if at least one), mean (SD)	1.1 (0.3)	1.1 (0.4)	1.2 (0.4)	*F*_2,4467_=13.6, **P*<.*001	1 and 3, 2 and 3
Having at least one comment, n (%)	663 (2.03%)	1088 (5.27%)	489 (10.06%)	χ^2^_2_=909.6, **P*<.*001	All groups
Number of comments (if at least one), mean (SD)	1.2 (0.6)	1.3 (0.9)	1.4 (1.1)	*F*_2,2237_=15.1, **P*<.*001	1 and 3, 2 and 3

### Changes in Healthiness Ratings

Among the 4863 Active users who had at least one valid picture as their first or second picture, 481 (9.89%) had a significant positive trend in healthiness scores. These “Improvers” differed from other Actives by having a higher total number of pictures (mean 126.68, SD 183.73 vs mean 51.41, SD 82.16; *t*_4861_=16.19; **P*<.*001), a longer usage period in days (mean 68.17, SD 42.95 vs mean 44.16, SD 36.34; *t*_4861_=13.54; **P*<.*001), and a higher number of pictures per day (mean 1.80, SD 1.70 vs mean 1.55, SD 1.58; *t*_4861_=3.25; **P*=.*001). In other words, they used the app for a longer time and did dietary self-monitoring more frequently.

Users with Strict diets had higher healthiness scores than users in other dietary preference categories and they also had the highest proportion of Improvers (Table 8).

**Table 8:** Average healthiness rating and number of users (Actives) that had a significant linear coefficient in their healthiness rating in each dietary preference category.

Scores/users	1. Not defined (n*=*732)	2. Everything (n*=*3023)	3. Strict (n*=*896)	4. Other (n*=*212)	Test statistics	Differences in post hoc comparisons
Average healthiness rating (first picture), mean (SD)	0.54 (0.19)	0.53 (0.19)	0.60 (0.18)	0.56 (0.18)	*F*_3,4859_=29.3, **P*<.*001	1 and 3, 2 and 3, 3 and 4
Average healthiness rating (all pictures), mean (SD)	0.56 (0.08)	0.57 (0.09)	0.63 (0.08)	0.60 (0.10)	*F*_3,4859_=149.8, **P*<.*001	All groups
Number of Improvers, n (%)	55 (7.51%)	281 (9.30%)	125 (13.95%)	20 (9.43%)	χ^2^_3_=22.5, **P*<.*001	1 and 3, 2 and 3
Number of Decliners, n (%)	14 (1.91%)	72 (2.38%)	32 (3.57%)	6 (2.83%)	χ^2^_3_=5.4, **P*=.*15	None

## Discussion

### Overall Adherence and Changes in Healthiness Ratings

Almost 190,000 people downloaded the app, The Eatery, between October 2011 and April 2012, but attrition was very high: less than 3% were active users, that is, used the app for more than a week and took 10 or more food pictures. Most of the users did not take any pictures (69%) or took only one picture (17%), which means that they only downloaded the app and experimented with it once without starting dietary self-monitoring. This is similar to most free apps, which are easy to join and try out even if there is no serious intention or commitment to start using the app [13,25]. Given the short usage period for majority of the users, many probably tried out the application for fun.

The Eatery was not marketed as a weight loss app but instead as a method to eat healthier (“Stop counting calories, start eating better”), and hence may have attracted a large number of users with no real interest in dietary improvements and thus lacking motivation for dietary self-monitoring. However, the few active users used the application on average 1.5 months. Dietary self-monitoring for this amount of time would be enough to lead to increased awareness of eating habits and changes in behavior, if done diligently. The average healthiness rating of all pictures was 0.58, slightly above the midpoint on the scale of 0 (“fat”) to 1 (“fit”). Hence, users did not photograph only healthy foods and there was room for improvement. A positive trend in healthiness ratings was still observed among only 10% of active users (0.3% of all users). Even if we assume that this trend reflects changes in their real-life eating behavior, the impact of the app on eating choices (or choosing which foods to record) appears to have been very small. Active users took less than two pictures per day on average, which means that a large portion of their eating was left unrecorded. Thus, the positive trend among some users could also mean that they started “gaming the system” by selectively photographing their foods to get better ratings and comments.

### Initiation of Self-Monitoring

Users who used the app for the first time on weekdays (especially on Tuesdays or Wednesdays) and during morning or daytime became semi-active or active users more often than those who started using the app during evenings or weekends. People’s varying eating patterns that depend on their schedules during workdays and outside work [34] can help explain this finding. Prior studies have found that diet quality is generally poorer during weekends than on weekdays and calorie intake is higher, especially in the form of fat and alcohol [31,32,35], so people do not necessarily want to start tracking their eating at these times. Moreover, people are generally less work-oriented in the evening and during weekends and may try out different apps just for fun. In contrast, someone who downloads a healthy eating app in the middle of the week during daytime probably has the intention to start keeping track of their eating. This time period could also be a fruitful time to suggest initiation of lifestyle changes, although everyone does not have the same work schedules.

The dietary preferences reported on the initial use were also connected to adherence level. Users who reported a “strict” diet (low fat, low/no carbs, or vegan/vegetarian) were most likely to become active users. They also gave 21% of all ratings, although only 9% of all users belonged to the strict group. Hence, it is possible that users with strict diets were already most interested in healthy eating. This is also supported by their healthiness ratings: active users with strict diets had higher average healthiness ratings for their first picture and also higher average healthiness ratings for all pictures than users in other dietary preference groups.

The motivation of sustained users might already be seen on the initiation of self-monitoring by looking at how much time and cognitive capacity they devote to it. This is supported by the finding that more than half of the active users (53%) gave a textual description for their first picture whereas less than one-fifth of the non-active users (18%) did so. In addition to pre-existing intention to start dietary self-monitoring, the initial user experience of the app most likely influenced the users’ intention to continue using it. Positive feedback received from peers for the first picture taken by the user was associated with higher adherence; active users had higher average healthiness ratings for the first picture than less active users. This begs the question—did they happen to take a picture of a healthy food and were encouraged by the good feedback to continue using the application or were they already healthy eaters, thus naturally photographing a healthy food? Because a higher proportion of active users also used the app for the first time during weekdays and daytime, the food that they chose to photograph first was probably their workday lunch, which is often healthier than foods that are eaten during weekends [35]. Hence, the timing of the initial use of the dietary self-monitoring app may be important both in terms of the users’ pre-existing motivation and the type of reinforcing feedback generated by the app.

### Peer Feedback

Although active users obtained more comments and likes for their pictures from peers than those who took only few pictures, the total percentage of pictures with comments (4%) and likes (15%) was quite low. Thus, most users had no connection to other users other than receiving and giving anonymous ratings. The social network formed in such a way is very loose: users neither know whose pictures they rate nor have any knowledge of who rates their pictures. The app itself did not offer explicit advice on what to do to improve eating habits or what constitutes a healthy diet. It may be that if users are merely told that their meal is unhealthy but not given any advice on what to do to make it better, they do not get enough value out of the experience and subsequently lack motivation to continue using the app [36]. People may also have very different ideas about what healthy or “fit” food is, but these differences do not seem to have influenced adherence in this study. Although the difference between the user’s own rating and average peer ratings was highest among active users, in practice this difference was very small.

An app like this relies on its users to provide one of its core functions, that is, peer feedback. It would be interesting to study what motivates people to participate in this crowdsourcing activity of giving ratings to others. One explanation is the reciprocity of the action: when a user rates someone else’s pictures, they also get ratings for their own. However, engaging in this activity for a long time might require an existing community or formation of stronger ties between users [27].

### Limitations and Challenges

The most significant limitation of the study is the lack of information about user demographics, behavioral outcomes, and initial motives. For example, the association between outcomes and adherence to dietary self-monitoring has been found to differ between race and gender groups in weight loss interventions [12], and it would have been interesting to see if similar patterns had emerged in this context. In this study, the application only provided data about the users’ dietary preferences. The general statistics about smartphone users in early 2012 suggest that iPhone users were slightly older than Android users and downloaded more apps in a month than users of other smartphone systems [37]. Users of The Eatery owned an iPhone so it is possible that their characteristics followed the same pattern. Collecting comprehensive data about users’ background may be challenging in free apps, which aim for fluent user experience, but creative ways to gather data such as asking one question per usage occasion could be devised in further studies.

The reliability of healthiness ratings is questionable because they were entirely crowdsourced. The idea of crowdsourcing is to take an average of many individuals’ estimates resulting in an estimate that can be surprisingly close to the truth, although individuals’ separate values may lie far from it. Crowdsourced ratings can be biased, resulting from cultural differences [26] or rater’s own food preferences. When pictures are rated as in this study, the quality of the picture is also likely to make a difference. Portion size estimation is difficult even when measurement aids are present in the picture [18,19,38]. Moreover, users did not photograph everything they ate so there is no way of knowing how healthily people ate in general and whether the observed positive trend in healthiness ratings among 10% of active users meant anything in practice.

Dietary decisions are often unconscious and affected by environmental factors more than people believe [39]. In this study, the location information of where the pictures were taken was available, but was not exploited, although dietary behaviors are likely to be linked with locations. In a recent study [40], volunteers used Twitter to report their food and were encouraged to add a photo and contextual information, such as company, mood, and reasons for eating. The data was used visualize the relationships between dietary and behavioral factors.

Some updates were released to the app during the six-month timeframe of data collection. These updates consisted of minor modification and fixes in the user interface of the app. They may have had a minor influence on the user experience of the app, but they were not included in the analyses since the main features and functions remained the same.

Finally, the app utilized self-regulation techniques of self-monitoring and feedback, but lacked other techniques derived from control theory [4,5]. The app did not prompt users to set specific goals or review behavioral goals and only implicitly prompted intention formation (“eat healthy”). Implementing these techniques could have given users a more specific purpose for using the app. Practical advice for healthy eating could also have been given by formation of implementation intentions through if-then planning, which has been shown to increase effectiveness of healthy eating interventions [10].

### Conclusions

As with most mobile apps, the majority of users tried the dietary self-monitoring app only once. Adherence was higher among users who had diets that were likely to restrict at least some unhealthy foods, and these kinds of users were also more active in rating other users’ foods. This could mean that this kind of an application attracts users with special diets and/or those interested in food. Moreover, initiation of self-monitoring in the middle of the week during daytime and the amount of feedback from peers were connected to higher adherence.

Even though the findings show that the app reached a large number of people, its actual impact among users remained small because most did not even start dietary self-monitoring with the app. If people would use the app as intended for dietary self-monitoring on a regular basis, they could experience some benefits through heightened awareness of their eating habits. Still, the app did not implement all self-regulation techniques that could have strengthened its impact and it lacked means to track changes in eating behavior systematically. Reaching those users who could benefit the most from dietary self-monitoring and maintaining their adherence remains a challenge.

## Abbreviations

ANOVA: analysis of variance
